# Towards equitable partnerships in global health research: experiences from Ethiopia, Uganda, Lao PDR and Vietnam

**DOI:** 10.1136/bmjgh-2025-019130

**Published:** 2025-06-30

**Authors:** Kassahun Alemu, Della Berhanu, Anna Bergström, Dinh Phuong Hoa, Stefan Swartling Peterson, Bounfeng Phoummalaysith, Goran Tomson, Rhoda Kitti Wanyenze, Lars Åke Persson

**Affiliations:** 1 University of Gondar, Gondar, Ethiopia; 2 Disease Control, London School of Hygiene and Tropical Medicine, London, England, UK; 3 Department of Women’s and Children’s Health, Uppsala University, Uppsala, Sweden; 4 Viet Nam Pediatric Association, Hanoi, Vietnam; 5 Department of Health Policy Planning and Management, Makerere University School of Public Health, Kampala, Uganda; 6 Department of Global Public Health Sciences, Karolinska Institutet, Stockholm, Stockholm County, Sweden; 7 Ministry of Health, Warsaw, Poland; 8 Department of Learning, Informatics Management, and Ethics, Karolinska Institutet, Stockholm, Sweden; 9 Department of Disease Control and Environmental Health, Makerere University School of Public Health, Kampala, Uganda

**Keywords:** Global Health, Interdisciplinary Research, Health policy, Public Health

## Abstract

Equitable partnerships in global health research can counteract power imbalances in this field. Theoretical perspectives have been provided on equitable global health partnerships, but there are few reports from researchers actively engaged in such partnerships. This article departs from the experiences of four long-term global health research partnerships, two in Africa and two in Asia. It describes the challenges in enhancing an equitable research partnership and how these were addressed. The examples illustrate that funders can play a pivotal role in counteracting power imbalances by assigning leadership roles and directing primary funding to institutions where the research occurs. Such a transition requires adaptations and a new mindset on both sides. Embedded research capacity enhancement, part of all four partnership examples, is essential in correcting power imbalances. Capacity enhancement should preferably include enhancing the broader research ecosystem within the partner university, across academic institutions in the country or beyond. The development of mutual trust and respect takes time and requires long-term engagement, transparency in budgeting, project planning and all steps of the research process. Reciprocity in learning is important for all partners. It may include twinning of research students, joint degrees and efforts to bring research findings into policy and practice in all partner contexts. The partnership examples illustrate the achievements and challenges in bringing new research evidence into policy and practice, where early involvement and continuous buy-in by policymakers are crucial.

**SUMMARY BOX**

- Equitable partnerships in global health research are crucial to meet the complexity of the emerging multiple crises and provide potential solutions.
- Theoretical perspectives on equitable partnerships have been provided, while there are few reports from researchers actively engaged in such partnerships.
- Based on experiences from four long-term partnerships in global health research, in Ethiopia, Uganda, Lao PDR and Vietnam, we relate the achievements and challenges in enhancing equitable partnerships in these contexts.
- Funders played important roles in establishing and supporting equitable research partnerships.
- Essential characteristics were reciprocity, inclusion, mutual trust and embedded capacity enhancement.
- These examples also illustrate shifting power dynamics; the lead institution and main grant recipient were now found where the research and training took place.

## Background and rationale

 The urgency of the current grand health challenges, which transcend national borders, necessitates global health research partnerships and collaborative action.^1^ Climate change, pandemic threats such as COVID-19, and conflict exacerbate existing challenges and create new ones.^2^,^4^ The essential role of global health research partnerships in addressing these challenges is more than just a truism since the emerging political tendencies of avoiding entanglements with other countries underline the importance of enhancing such collaborations. The Sustainability Development Goal 16 focuses on the essential role of strong institutions, and Goal 17 emphasises the role of partnerships in these efforts.

Resources and capacity for global health research are unequally distributed across continents and countries.^5^ However, the potential impact of equitable global health research partnerships in compensating for these imbalances is significant, counteracting neocolonial relationships and enhancing relevance in the research process from problem formulation to research output and policy formation.^6^ Equitable research partnerships may be characterised by mutual participation, mutual trust and respect, mutual benefit and an equal value placed on each partner’s contribution at all stages of the research process.^7^

Several theoretical frameworks and models for equitable research partnerships have been suggested. In 2021, Schrieger *et al* provided a theoretical model with transactional, collaborative and transformational levels as the foundation for a successful and equitable global health partnership.^8^ The transactional components were governance, resources and expertise, power management, transparency and accountability, data and evidence, respect and curiosity. Shared vision, relationship building, understanding and trust made the collaborative level. The transformational level was built by equity and sustainability.

Components and levels were built on the previously mentioned dimensions.^8^ Zaman *et al* suggested that creating an equitable partnership in global health research may be based on four pillars: co-creation, communication, commitment and continuous review.^6^ Cross-cutting values of fairness, respect, care and honesty reinforce the pillars. The ESSENCE on Health Research and UK Collaborative on Development Research Good Practice Document presented barriers and enablers of equitable research partnerships.^9^ The focus was mainly on the software of partnerships, providing four approaches to enhance equitable partnerships. These approaches emphasised supporting the partnership ecosystem, addressing power imbalances and monitoring and learning from successes and failures. They also included understanding context and culture, investing in research management and budgeting for partnership development, including direct funding to the partner institutions and researchers where the research was done. The approaches also included governance and investing in long-term, sustained partnerships.

Thus, while several theoretical perspectives on equitable global health partnerships have been provided, more research reports are needed from researchers actively engaged in such partnerships. Voices from Europe and the USA have dominated the discussion on equitable partnerships.^10^ However, it is crucial to emphasise that practical experiences addressing challenges of power imbalances are essential and necessary in the ‘decolonizing’ efforts toward equitable partnerships.^11^

This article is presented in narrative form, departing from the experiences of four long-term global health research collaborations in different contexts and research fields, where the strive for equitable partnerships has been central. It is based on repeated discussions across the group of authors and aims to describe the challenges in enhancing an equitable research partnership and how these were addressed. We relate the context-specific experiences from four settings of developing equitable partnerships to meet current and future global health challenges.

## Four country examples

Below, we provide four examples of long-term partnerships, selected to represent different research fields and contexts, two in Africa and two in Asia. The characteristics, challenges and achievements are presented and commented on.

### Ethiopia: evaluations with embedded research capacity development

Based on requests from the Ethiopian Ministry of Health, a partnership between the Ethiopian Public Health Institute, several Ethiopian universities and the London School of Hygiene and Tropical Medicine (LSHTM) has been working and learning from each other in health system evaluations and implementation research combined with capacity development during the last 10 years.^12^ The overall intention was to stimulate and develop the capacity of Ethiopian universities to respond to the Ministry’s need for evidence for policy and planning. The Bill & Melinda Gates Foundation initially funded these projects with grants to LSHTM that provided subawards to the Ethiopian partners. The latest grants were provided to the University of Gondar and Addis Ababa University with a side grant or subaward to LSHTM. Throughout 10 years of partnership, an LSHTM team has been based at the Ethiopian Public Health Institute, Addis Ababa. This arrangement substantially enhanced equity in the partnership through the day-to-day collegiality, the gradual building of mutual trust and the growth of a shared vision for the joint work. The partnership has included various projects; a few are mentioned. In the Dagu project, the evaluation of an intervention to enhance the utilisation of child curative services in four major regions was combined with the training of nine PhD students from Ethiopian universities, the public health institute and Regional Health Bureaus.^13^ These students were registered at Ethiopian universities and jointly supervised by senior colleagues from the Ethiopian university and the UK partner institution. They focused on topics that would enrich the overall evaluation, such as quality of care, equity and the context for the primary healthcare workers.^14^,^16^ In the Operational Research and Coaching for Analysts (ORCA) project, operational research on data from the country’s routine health information system was combined with the coaching of 38 analysts from the ministry of health and linked institutions.^17^ The analysts planned and performed field studies of the generation, use and quality of the health system routine data.^18^ The Lihiket (excellence) project was built on experiences from previous studies. The Vitae Research Development Framework was used to assess and prioritise capacity development needs, with comprehensive representation from partner institutions.^19^ Based on the capacity needs assessment, institutional capacity development efforts included training opportunities in implementation research, community-based trials, grant applications, project management and communication of research results. The activities were built on a learning philosophy at the University of Gondar, where ‘360-degree learning’, that is, reciprocal learning and equal partnership, was central. These exercises resulted in a training agenda, where the complementarity of expertise from partner institutions was used with documentation of modules and materials for future use, which facilitated the sustainability of the activity. The project also included training of postdoctoral fellows, who were group-mentored by senior partners, realising the importance of mentoring for career development and capacity development.^20^ Based on interactions with maternal and child health representatives at the ministry of health, the fellows developed policy-relevant research based on data from recent national surveys. As a result, postdoctoral training has been institutionalised in the public health school at the University of Gondar. In recently initiated joint projects, such training opportunities have been further expanded to five other universities.

### Uganda: joint PhD degree partnership

Enabled by a grant from the Swedish International Development Cooperation Agency’s research capacity development division, Makerere University and Karolinska Institutet (KI) embarked on a research capacity enhancement collaboration with a focus not just on the individual student but also on the supervisor capacity and the research support system at Makerere. Makerere University identified the research areas and group leaders to which relevant KI researchers were added to write formal project applications to the funder, which Makerere submitted. All projects were peer-reviewed. Successful projects twinned supervisors from the two universities to supervise Makerere PhD students. The collaboration also enrolled Karolinska PhD students on separate funding. This arrangement served to lessen power imbalances in project formulation and enabled a collaborative spirit and mutual support at both supervisor and student levels, as Makerere-KI research groups examined a diverse set of topics from mental health to acute febrile illness management in health systems.^21^ Initially, all research students were enrolled only at Makerere University, which was a disincentive for early to mid-career KI researchers to co-supervise since the KI incentive system favours graduating students from KI. This issue was one of the reasons for developing a ‘double PhD degree’ agreement, whereby the student was enrolled at both universities and satisfied both universities’ graduation requirements. The PhD candidate was examined by a jointly appointed examination committee, getting one diploma with two logos. While administratively cumbersome, this double degree became the favoured option for over 50 students. This degree collaboration changed Makerere’s examination routines from closed room monograph examination to public defence examination of degrees by coursework and published papers, most of which had Makerere’s PhD students as first authors.^21^

The funding, spanning over two decades, yielded numerous scientific outputs and significantly influenced national^22^ and global policies. For instance, it played a pivotal role in shaping the malaria treatment policies in Uganda and the WHO/UNICEF Integrated Community Case Management of febrile illness in children, a strategy now being implemented across Africa and parts of Asia.^23^ The enhanced capacity to attract and manage grants secured additional funding for joint applications. Even after the end of the Swedish International Development Cooperation Agency’s funding, the collaboration remains active and robust. It evolved into a jointly owned and equitably directed Centre of Excellence for Sustainable Health.^24^ This centre is actively benefiting countries in the region, including universities in the Democratic Republic of Congo and Somalia.^25^

Central to the success of this 25-year collaboration was the cultivation of a shared vision and trust among project leaders at all levels. Physical mobility, meetings and reciprocal exchange of staff and students were instrumental in this process. The collaboration has significantly enhanced the research system not only at Makerere but also at KI. A prime example of reciprocal learning is stroke rehabilitation via mobile phone support, initially developed for Ugandan use, now being transferred to Sweden for evaluation in the Swedish healthcare system.^26^

### Lao People’s Democratic Republic: research evidence in pharmaceutical policy development and implementation

In 1991, the Lao government sought assistance from the Swedish International Development Cooperation Agency to address issues in the pharmaceutical sector. The initial problem was the prevalence of fake and substandard drugs. A review team, comprising the KI, the Lao Ministry of Health and two experts on pharmaceutical drug regulation and use of medicines from Thailand, took a comprehensive approach.^27^ They proposed intersectoral action to develop a National Drug Policy, which involved representatives from six ministries, various professional groups and Non-Governmental Organisations (NGOs). Small-scale health system surveys focusing on households, prescribers, pharmacies and drug cost comparisons were conducted with methodological support from a Thai epidemiologist to diagnose problems that helped develop the National Drug Policy, which was launched in 1993.^27^ The development was swift, but the implementation could theoretically have been faster. The partners introduced health policy and systems research as institutional research capacity enhancement and to support the implementation of the National Drug Policy. Lao researchers, policymakers and practitioners set research priorities. An international multidisciplinary team led by the Lao National Institute of Public Health and KI supported the programme with a total of 15 projects covering the different elements of the National Drug Policy, such as pharmaceutical regulation, private pharmacy practice, the quality of drugs, drug therapeutic committees, case management of malaria, diarrhoea and pneumonia at hospitals, and community perceptions. These projects involved problem identification, design, data collection, analysis and report writing.^28^ Mixed methods were used, including qualitative studies combined with questionnaires^29^ and cross-sectional studies using Good Pharmacy Practice guidelines operationalised into performance indicators.^30^ The effectiveness of standard treatment guidelines on the quality of case management at provincial hospitals was evaluated by a cluster-randomised trial.^31^ By 1998, the research and the generated findings were an integrated part of the National Drug Policy process. The approach was appreciated by national and provincial decision-makers.^32^ The partnership implied reciprocal learning and capacity building, including 20 international publications on themes decided by the Lao colleagues and with balanced coauthorship. To mitigate power imbalances, capacity building also included English language training, managerial issues and efforts to strengthen institutions in their implementation of the National Drug Policy. Success factors included a shared vision, trust, clear objectives, funding for more than 10 years, Lao political support and strong dual leadership. The 2004 World Health Report ‘Knowledge for Better Health’ pointed out the Lao National Drug Policy as one of two examples of how research can strengthen the health systems.

### Vietnam: community and health facility engagement interventions for neonatal survival

The starting point for this partnership on neonatal health and survival in Vietnam was an analysis of under-five and neonatal mortality in a Red River Delta area from the 1970s to 2000, showing a marked reduction of under-five mortality but persistently high neonatal mortality.^33 34^ This partnership of Vietnamese and Swedish academic institutions was built on having a shared goal, trust in each other’s competencies, transparent administrative processes, financial management and curiosity of the partnering institutions. The partnership was funded by several external grants, including funding from the Department of Science and Technology in the study province and pro bono contributions from Vietnamese institutions and health facilities.

The problem formulation, intervention and evaluation designs were allowed to take time, involving discussions with the Ministry of Health, regional health authorities and the nationwide Women’s Union. Partners gradually added complementary competencies, always with a deep respect for the local culture and understanding its importance in developing an effective intervention. The team was keen to develop an intervention that was not only effective but also culturally acceptable and based on existing groups for collaboration across different sectors. The research offered opportunities for reciprocal learning, including the twinning of PhD students from Swedish and Vietnamese institutions. The project was named NeoKIP, neonatal knowledge into practice, and evaluated by a cluster-randomised trial in Quang Ninh province, Vietnam. Laywomen recruited from the Women’s Union facilitated monthly meetings for 3 years in existing intersectoral groups composed of staff from health centres and critical persons in the communes, chaired by the vice chairman. A problem-solving approach was employed, and many local problems related to the mother-and-newborn dyad and maternity services were identified and addressed. The neonatal mortality rate was reduced by half after 3 years,^35^ an effect sustained when followed up 6 years from the initial start of the intervention.^36^ The partnership team was aware that this approach had limited or no impact on problems within health facilities, and in a proof-of-concept study, the engagement intervention included both community groups and problem-solving groups at health facilities.^37^ Despite the intervention’s sustained effect on neonatal survival, the actions’ limited additional costs and the involvement from the start by national and regional policymakers, there was no scale-up of the intervention in Vietnam. Several factors, including the lack of inclusion of the participatory approach in national and regional perinatal health guidelines, could have caused this problem.

## Developing equitable partnerships

We have briefly described four diverse long-term partnerships in global health research: two in Africa and two in Asia. The characteristics and experiences in enabling equitable partnerships across the four examples are briefly shown in table 1. We selected partnership examples with different characters. The Ethiopian collaboration was created by the Ministry of Health, the funder, Ethiopian academic institutions and the UK partner to evaluate interventions and perform implementation research combined with research capacity development efforts. In Uganda, the funder invited the Ugandan university to collaborate with Swedish partners to develop individual and institutional research capacity, create a critical mass and generate policy-relevant evidence. The Lao example was focused on research evidence into policy: partners generating research evidence, leading to the development and implementation of a national drug policy. In Vietnam, partners collaborated in implementation research on community engagement to reduce neonatal mortality.

**Table 1:** Brief characterisations of transactional, collaborative and transformational levels in the development of equitable partnerships in Ethiopia, Uganda, Lao PDR and Vietnam

Components	Partnership			
	Ethiopia	Uganda	Lao PDR	Vietnam
Transactional level				
Funding	Funding of combined research collaboration and capacity development. Transition towards primary funding to the Ethiopian institutions and side grant or subaward to the UK partner.	20 years of funding from the Swedish International Development Cooperation Agency for research and institutional and individual capacity development created a critical mass of researchers in several fields.	After initial major funding uncertainties, partners finally secured 10 years of funding followed by another 5 years of continued university collaboration.	Several funders were needed to secure partnership and capacity development over time, including Vietnamese pro bono contributions.
Governance, administration	The Ethiopian partner employed a skilled project administrator who enhanced the institution’s administrative capacity and has been an effective interpreter between the different administrative cultures. The transition towards leadership on the Ethiopian side and ‘allyship’ for the UK partners implies adaptations on both sides.	Supervisors from Uganda and Sweden were twinned to supervise each PhD student. One academic coordinator on each side worked together to ‘troubleshoot’ issues as they arose.	The project was led by a team from Karolinska Institutet and Department of Pharmacy, Lao Ministry of Health, in consultation with the funder. Initial power imbalances, partly explained by language barriers. Over time, development towards equitable partnership and full Lao project ownership.	Projects were jointly governed and administered by teams from Sweden and Vietnam; the latter representing the National Children’s Hospital and the Department of Maternal and Child Health at the Ministry of Health.
Expertise, complementarity	A culture of complementarity was nurtured across partner institutions, early career and senior researchers and across administrative and research activities.	Ugandan supervisors and students had unrivalled contextual experience, while Sweden counterparts had more resources at their disposal. Together that made strong teams.	The project involved six Lao ministries, the National Institute of Public Health and a team including expertise in law, medicine, pharmacy and behavioural science.	The team gradually added expertise, seeking complementarity across partner institutions.
Transparency, respect, accountability	Cultivated transparency and mutual respect in all planning, budgeting, performance and follow-up by joint leadership, supervision and frequent Internet-based meetings.	Trust comes from transparency and clear procedures. Applications for funding were written jointly and submitted by Makerere.	Mutual respect and transparency in planning, priority setting, budgeting, joint leadership, regular physical meetings.	Transparency in planning, co-production, valuing different perspectives.
Shared data, evidence	Data sharing agreements, joint work with data management, analyses and reporting. Equitable plans for publications and communication of results.	Institutional review boards in Uganda reviewed all protocols. All data were accessible to both Ugandan and Swedish principal investigators. PhD students were first authors on all papers, co-supervisors alternated as senior authors.	Health Systems Research principles were used in the 15 projects including Lao teams with policymakers, practitioners, researchers in priority setting, designing, data collection, analysis and report writing.	Joint work in all steps with data and analyses. Publication plans agreed and continuously revised. Authorship priority to PhD students, fair distribution of authorship across partners.
Collaborative level				
Shared vision	The vision for the collaborative studies was initiated in discussions between the Ministry of Health and the funder, and further developed by universities, regional health authorities and the UK partner.	Developing research capacity in both partners and institutions to contribute to the betterment of health through improved policies and practice.	In the collaboration between the multidisciplinary Lao and Swedish teams in discussion with national and regional Lao colleagues, a shared vision was developed to generate a drug policy based on research evidence.	Long time and repeated discussions to develop a shared vision for the partnership and research plans.
Building relationship, trust	A team from the UK partner institution was embedded at an Ethiopian institution, working shoulder to shoulder, having frequent brief meetings.	Trust was built by the long-term relationship and personal friendships. Appointment of faculty in the other institution and exchange of teachers and students.	A deliberate attempt from the beginning to learn and be respectful to the Lao context as well as underlining the reciprocal learning and usefulness also for the Swedish team and Karolinska Institutet.	Long-term relationship, mutual benefits increased trust and understanding. Swedish researchers and research students were embedded into the Vietnamese institutions.
Embedded research capacity development	PhD and postdoctoral training and institutional capacity development was part of the research collaboration.	Research questions were developed from knowledge and practice gaps in Uganda and answered in actual health-system settings.	Embedded efforts to enhance the research capacity ranged from English language training to advanced engagement of the partners in health systems research.	Several PhD students from both partners twinned into the project, with a focus on key aspects of the planned research.
Transformational level				
Enhanced research ecosystem	The partnership stimulated collaboration within and between Ethiopian universities and institutions and regional health authorities.	Research systems were built, including health and demographic surveillance site and multidisciplinary research groups.	In these efforts, academic institutions, government institutes, agencies and ministries developed a shared vision and approach to generate a research-based drug policy.	Communities, health centres, hospitals, health authorities and academic institutions worked together.
Evidence to policy	Several examples of research findings influencing policy and practice, especially the primary healthcare transformation and routine data quality efforts.	Several examples of research findings to policy in Uganda and beyond.	The ‘Evidence-based Lao National Drug Policy’ used Health System Research from the beginning to shape and implement the policy.	Despite the involvement of a wide range of stakeholders and project success (reduced neonatal mortality), no scale-up and policy change. More attention should have been given to policy-level buy-in.
Reciprocity	The long-term partnership enabled involved researchers to grow and develop their careers.	Mutual training opportunities, research findings in Uganda brought to Sweden and implemented.	Reciprocity was part of the collaboration from the very beginning.	Twinning of PhD students from both partners created reciprocal benefits, increased trust and understanding.
Sustainability	The emphasis on individual and institutional capacity development has created a sustained impact.	Next generation research leaders and groups were brought up. The PhD programme was established at Makerere and grants office capacity development.	The Lao National Drug Policy and its components, such as the standard treatment guidelines, still exist and have been revised after the end of the project.	The effect of the intervention had a sustained positive effect on neonatal survival in the region. The former research students continued into lead positions.

The three levels and components modified after Schriger *et al*, 2021.

In these four partnerships, the focus was equity in contrast to equality. The point of departure for an equitable partnership is to analyse the financial and academic resources needed to counteract current power imbalances and reach the agreed goal.^38^ The Ethiopian and Ugandan examples illustrate that research funders can initiate and support equitable research partnerships and play a crucial role in addressing such power imbalances (table 1). Their ability to invest for the long term, set up rules for equitable budgets, show that they value complementary expertise across partners, and allocate funds directly to the institution where the research is done enhances the equity and sustainability of these partnerships. In this way, funders can enhance strong dual leaderships of long-term collaboration. In contrast, funders did not play any role in influencing partnership characteristics in the Vietnam case—the development of an equitable partnership was based on long-term professional relations and mutual trust.

So far, European or US partner institutions largely have had the lead roles and main grants for projects in Africa or Asia. As illustrated by the Ethiopian and Ugandan examples, there is now a trend in which funders prefer the logical choice of providing the lead role and main grant to an institution where the project and training are implemented. This shift also implies that the new lead institutions must further develop their project management and administration capacity. Some described partnerships invested in administrative staff and developed training packages to meet this need. The collaborating institutions from Europe to the USA also need to develop their ability to ‘allyship’—a transition that may demand administrative adaptations, modified rules for promotion and a new mindset.^39^ In the examples from Ethiopia, Uganda and Vietnam, staff from the European side were based at the local partner institutions, increasing mutual understanding and making joint decisions and adaptations easy.

Embedded research capacity development, which refers to the continuous and integrated process of building research skills and knowledge, is essential in correcting power imbalances and enabling equitable partnerships.^12^ It is also pivotal for evidence-informed policies as exemplified by the Lao National Drug Policy case.^28^ Depending on the need, capacity-building efforts may include individual training and institutional components on different levels (table 1). In two of the partnership examples, there were postdoctoral training opportunities. In many African and Asian settings, opportunities are few for early career researchers to gain more experience in research proposal writing, leading and managing research projects and other knowledge and skills needed in the research process. Mentoring these early career researchers may contribute to their career planning and create a critical mass of independent researchers.^40^ These four partnership cases also provide examples of enhancing the broader research ecosystem within the partner universities, across several academic institutions in the country or with partners in the region. A central component in an equitable partnership is reciprocity in learning, as illustrated in the case examples with twinned PhD students, joint degrees^21^ and the generation of new evidence of relevance for all partners and their contexts.

In these examples, the risk of brain drain from Africa or Asia to Europe has been mitigated by two approaches. First, most individual and institutional capacity development activities have taken place in Ethiopia, Uganda, Lao and Vietnam and not at the European partner institutions. Second, the PhD or postdoctoral training offered at the European institutions has been based on a ‘sandwich’ design with most time spent at their home institutions and their research focus integrated into the larger partnership priorities.

These cases also illustrate the importance of engaging local stakeholders and communities in research. Such engagement requires formalising roles and identifying a shared goal—a research question or a focus of the effort the partnership aims to contribute. In the Vietnamese example, the shared questions revolved around improving neonatal survival within established structures and how the participants, with their diverse capacities, could contribute to studying and communicating the efforts made.^37^ In the Lao case, the development of the National Drug Policy was remarkably swift. At the same time, the implementation was slow until the stakeholders identified implementation research priorities and participated in these activities.

The development of mutual trust and respect takes time. Transparency in budgeting and other parts of the project planning, and mutual participation and benefit in all parts of the research process are essential ingredients. Fair data-sharing agreements and authorship rules must also be established.^41 42^

The four country examples illustrate evidence generation that led to policy and practice changes. Early involvement of policymakers and continued buy-in into the research process are essential. Even so, the pathway from research findings to policy may be complicated.^43^

We are aware of the fabric of inequities in global health knowledge production that have been labelled epistemic injustice.^44^ Based on our lived experiences, we have mentioned some issues and challenges in enhancing equitable partnerships. However, in global health research partnerships, we carry a ballast of practices in all steps of the research cycle that create and sustain power imbalances in knowledge production. It is essential to raise awareness about these injustices to equitably meet current and future global health needs.

In this text, we have avoided the common dichotomies and crude classifications we often use to classify people, countries and regions.^45^ Languages such as developed and developing, high and low-income and global north and south tend to take hierarchical and inequitable relations for granted.

These practical experiences of research collaboration validate the theoretical frameworks cited in the background section, emphasising the role of funders in addressing power imbalances. While these four experiences involve policy-and-practice relevant research, the same characteristics of mutual respect and trust, explicit agreement on roles and responsibilities and transparency and accountability in governance are equally essential in other major global health investments and technology transfer.

## Conclusion

Power asymmetries are a significant challenge in global health research partnerships. They may include, but are not limited to, gaps in available financial resources and technical expertise.^46^ We have provided a few examples of long-term collaboration in global health research, where efforts to develop equitable partnerships have been central. Funders can be crucial in promoting, establishing and supporting equitable research partnerships. Scientific partnerships in global health research based on reciprocity, inclusion and mutual trust are needed to meet the complexity of the emerging multiple crises and provide potential solutions.^4^ However, if not set up as fair and equitable partnerships, they may reinforce power imbalances and be less effective.^47^ Equitable partnerships can transform global health collaboration from helping and educating others towards working in mutual trust and benefit.^48^

## Data Availability

Data sharing not applicable as no data sets generated and/or analysed for this study.

## References

[1] Koplan JP, Bond TC, Merson MH (2009). Towards a common definition of global health. Lancet.

[2] Mahase E (2022). UN warns of 'devastating' effect of covid-19, conflict, and climate change on women’s and children’s health. BMJ.

[3] Hendriks SL, Montgomery H, Benton T (2022). Global environmental climate change, covid-19, and conflict threaten food security and nutrition. BMJ.

[4] Kwamie A, Causevic S, Tomson G (2024). Prepared for the polycrisis? The need for complexity science and systems thinking to address global and national evidence gaps. BMJ Glob Health.

[5] Olufadewa II, Adesina MA, Ayorinde T (2020). From Africa to the World: Reimagining Africa’s research capacity and culture in the global knowledge economy. J Glob Health.

[6] Zaman M, Afridi G, Ohly H (2020). Equitable partnerships in global health research. Nat Food.

[7] UK Collaborative on Development Research (UKCDR) (2024). Equity in research partnerships. https://ukcdr.org.uk/priority-area/equitable-partnerships/.

[8] Schriger SH, Binagwaho A, Keetile M (2021). Hierarchy of qualities in global health partnerships: a path towards equity and sustainability. BMJ Glob Health.

[9] ESSENCE on Health Research and UKCDR (2022). Four approaches to supporting equitable research partnerships. https://tdr.who.int/publications/m/item/2022-09-07-four-approaches-to-supporting-equitable-research-partnerships.

[10] Rees CA, Rajesh G, Manji HK (2023). Has Authorship in the Decolonizing Global Health Movement Been Colonized?. Ann Glob Health.

[11] Finkel ML, Temmermann M, Suleman F (2022). What Do Global Health Practitioners Think about Decolonizing Global Health?. Ann Glob Health.

[12] Adegnika AA, Amuasi JH, Basinga P (2021). Embed capacity development within all global health research. BMJ Glob Health.

[13] Berhanu D, Okwaraji YB, Defar A (2020). Does a complex intervention targeting communities, health facilities and district health managers increase the utilisation of community-based child health services? A before and after study in intervention and comparison areas of Ethiopia. BMJ Open.

[14] Getachew T, Abebe SM, Yitayal M (2021). Association between a complex community intervention and quality of health extension workers’ performance to correctly classify common childhood illnesses in four regions of Ethiopia. PLoS One.

[15] Wuneh AD, Medhanyie AA, Bezabih AM (2019). Wealth-based equity in maternal, neonatal, and child health services utilization: a cross-sectional study from Ethiopia. Int J Equity Health.

[16] Getachew T, Abebe SM, Yitayal M (2021). Health extension workers’ perceived health system context and health post preparedness to provide services: a cross-sectional study in four Ethiopian regions. BMJ Open.

[17] Busza J, Lemma S, Janson A (2021). Strengthening routine health data analysis in Ethiopia: the Operational Research and Coaching for Analysts (ORCA) experience. Glob Health Action.

[18] Adane A, Adege TM, Ahmed MM (2021). Exploring data quality and use of the routine health information system in Ethiopia: a mixed-methods study. BMJ Open.

[19] Vitae (2024). Researcher development framework. https://www.vitae.ac.uk/researchers-professional-development/about-the-vitae-researcher-development-framework/developing-the-vitae-researcher-development-framework.

[20] Bonaconsa C, Nampoothiri V, Mbamalu O (2024). Mentorship as an overlooked dimension of research capacity strengthening: how to embed value-driven practices in global health. BMJ Glob Health.

[21] Sewankambo N, Tumwine JK, Tomson G (2015). Enabling dynamic partnerships through joint degrees between low- and high-income countries for capacity development in global health research: experience from the Karolinska Institutet/Makerere University partnership. PLoS Med.

[22] Nabyonga-Orem J, Ssengooba F, Macq J (2014). Malaria treatment policy change in Uganda: what role did evidence play?. Malar J.

[23] Awor P, Kalyango JN, Stålsby Lundborg C (2022). Policy Challenges Facing the Scale Up of Integrated Community Case Management (iCCM) in Uganda. Int J Health Policy Manag.

[24] Wanyenze RK, Alfvén T, Ndejjo R (2023). Sustainable health-a call to action. BMC Glob Public Health.

[25] CESH. Centre of Excellence for sustainable Health (2024). A collaboration between makerere university in Uganda and Karolinska institutet in Sweden. https://cesh.health.

[26] Ytterberg C, Eriksson G, Stefansdotter E (2024). Is e-health the future for stroke rehabilitation? health professionals’ experiences of implementation of a mobile phone-supported and family centred rehabilitation intervention after stroke in uganda. In Review.

[27] Paphassarang C, Tomson G, Choprapawon C (1995). The Lao national drug policy: lessons along the journey. Lancet.

[28] Jönsson K, Phoummalaysith B, Wahlström R (2015). Health policy evolution in Lao People’s Democratic Republic: context, processes and agency. Health Policy Plan.

[29] Paphassarang C, Wahlström R, Phoummalaysith B (2002). Building the national drug policy on evidence--a cross sectional study on assessing implementation in Lao PDR. Southeast Asian J Trop Med Public Health.

[30] Syhakhang L, Stenson B, Wahlström R (2001). The quality of public and private pharmacy practices. A cross sectional study in the Savannakhet province, Lao PDR. Eur J Clin Pharmacol.

[31] Wahlström R, Kounnavong S, Sisounthone B (2003). Effectiveness of feedback for improving case management of malaria, diarrhoea and pneumonia--a randomized controlled trial at provincial hospitals in Lao PDR. Trop Med Int Health.

[32] Tomson G, Paphassarang C, Jönsson K (2005). Decision-makers and the usefulness of research evidence in policy implementation--a case study from Lao PDR. Soc Sci Med.

[33] Hoa DP, Nga NT, Målqvist M (2008). Persistent neonatal mortality despite improved under-five survival: a retrospective cohort study in northern Vietnam. Acta Paediatr.

[34] Nga NT, Målqvist M, Eriksson L (2010). Perinatal services and outcomes in Quang Ninh province, Vietnam. *Acta Paediatr*.

[35] Persson LÅ, Nga NT, Målqvist M (2013). Effect of Facilitation of Local Maternal-and-Newborn Stakeholder Groups on Neonatal Mortality: Cluster-Randomized Controlled Trial. PLoS Med.

[36] Eriksson L, Nga NT, Hoa DTP (2018). Secular trend, seasonality and effects of a community-based intervention on neonatal mortality: follow-up of a cluster-randomised trial in Quang Ninh province, Vietnam. *J Epidemiol Community Health*.

[37] Bergström A, Hoa DP, Nga NT (2023). A facilitated social innovation: stakeholder groups using Plan-Do-Study-Act cycles for perinatal health across levels of the health system in Cao Bang province, Vietnam. Implement Sci Commun.

[38] Boum II Y, Burns BF, Siedner M (2018). Advancing equitable global health research partnerships in Africa. BMJ Glob Health.

[39] Pai M, Bandara S, Kyobutungi C (2024). Shifting power in global health will require leadership by the Global South and allyship by the Global North. Lancet.

[40] Mbouamba Yankam B, Kong JD (2023). Global Health Mentorship: Challenges and Opportunities for Equitable Partnership. BMJ Glob Health.

[41] Sam-Agudu NA, Abimbola S (2021). Using scientific authorship criteria as a tool for equitable inclusion in global health research. BMJ Glob Health.

[42] Evertsz N, Bull S, Pratt B (2023). What constitutes equitable data sharing in global health research? A scoping review of the literature on low-income and middle-income country stakeholders’ perspectives. BMJ Glob Health.

[43] Khomsi K, Bouzghiba H, Mendyl A (2024). Bridging research-policy gaps: An integrated approach. *Environ Epidemiol*.

[44] Bhakuni H, Abimbola S (2021). Epistemic injustice in academic global health. Lancet Glob Health.

[45] Khan T, Abimbola S, Kyobutungi C (2022). How we classify countries and people-and why it matters. BMJ Glob Health.

[46] Moon S (2019). Power in global governance: an expanded typology from global health. Global Health.

[47] Voller S, Chitalu C-CM, Nyondo-Mipando AL (2022). 'We should be at the table together from the beginning': perspectives on partnership from stakeholders at four research institutions in sub-Saharan Africa. Int J Equity Health.

[48] Sewankambo NK, Wallengren E, De Angeles KJC (2023). Envisioning the futures of global health: three positive disruptions. Lancet.

